# First Report on the Molecular Detection of Canine Astrovirus (CaAstV) in Dogs with Gastrointestinal Disease in Ecuador Using a Fast and Sensitive RT-qPCR Assay Based on SYBR Green^®^

**DOI:** 10.3390/vetsci11070303

**Published:** 2024-07-04

**Authors:** Anthony Loor-Giler, Sara Castillo-Reyes, Silvana Santander-Parra, Martín Campos, Renán Mena-Pérez, Santiago Prado-Chiriboga, Luis Nuñez

**Affiliations:** 1 Laboratorios de Investigación, Dirección General de Investigación, Universidad de Las Américas (UDLA), Antigua Vía a Nayón S/N, Quito EC 170124, Ecuador; anthony.loor@udla.edu.ec; 2 Facultad de Ingeniería y Ciencias Aplicadas, Carrera de Ingeniería en Biotecnología, Universidad de Las Américas (UDLA), Antigua Vía a Nayón S/N, Quito EC 170124, Ecuador; 3 Facultad de Ciencias de la Salud, Carrera de Medicina Veterinaria, Universidad de Las Américas (UDLA), Antigua Vía a Nayón S/N, Quito EC 170124, Ecuador; sara.castillo.reyes@udla.edu.ec (S.C.-R.); or silvanahsp@yahoo.com (S.S.-P.); rpmena@uce.edu.ec (R.M.-P.); or sdpch2021@gmail.com (S.P.-C.); 4 Facultad de Industrias Agropecuarias y Ciencias Ambientales, Carrera Agropecuaria, Universidad Politécnica Estatal del Carchi (UPEC), Antisana S/N y Av. Universitaria, Tulcán EC 040102, Ecuador; rolando.campos@upec.edu.ec; 5 Facultad de Ciencias Veterinarias, Universidad Nacional de Rosario (UNR), Boulevard Ovidio Lagos y Ruta 33 Casilda, Santa Fe 2170, Argentina; 6 Facultad de Medicina Veterinaria y Zootecnia, Universidad Central del Ecuador, Gatto Sobral y Jerónimo Leiton, Quito EC 170521, Ecuador; 7 Clínica Veterinaria Docente, Universidad de Las Américas (UDLA), Calle Shuara N40-55y Av. de Los Granados, Quito EC 170503, Ecuador; 8 One Health Research Group, Universidad de Las Américas (UDLA), Antigua Vía a Nayón S/N, Quito EC 170124, Ecuador

**Keywords:** canine astrovirus, qPCR, SYBR Green, first report

## Abstract

**Simple Summary:**

Enteric viruses are the main cause of gastrointestinal disease in dogs. Canine astrovirus (CaAstV) is an enteric virus that causes severe dehydration, diarrhea and vomiting, among other gastrointestinal symptoms. The present study is based on the design, standardization, and execution of a rapid and sensitive qPCR protocol using SYBR Green for the identification of CaAstV in dogs with gastroenteritis in Ecuador as a causative agent of gastrointestinal disease in co-infection with other viruses, such as canine parvovirus. The assay was shown to be repeatable, specific and sensitive based on the tests performed. The method was tested on 221 samples from dogs with gastroenteritis, of which 52.8% showed positivity for CaAstV. Sequencing of a segment of the ORF1b gene of the virus genome was performed to validate the presence of the virus, leading to the identification of two possible lineages of the virus circulating in Ecuador. This is the first report of CaAstV in the country. The present trial demonstrates the need for the application of molecular methods for the diagnosis and monitoring of emerging viruses in animals that could be influencing canine mortality due to gastrointestinal disease.

**Abstract:**

Enteric viruses are responsible for a significant number of gastrointestinal illnesses in dogs globally. One of the main enteric viruses is the canine astrovirus (CaAstV), which causes diarrhea in dogs of various ages. It is linked to symptoms such as diarrhea, vomiting, depression and a significant mortality rate due to gastrointestinal disorders. It is a single-stranded positive RNA virus, with three open reading frames, ORF1a, ORF1b and ORF2, where the last one codes for the virus capsid protein and is the most variable and antigenic region of the virus. The aim of this work is to develop and standardize a quick detection method to enable the diagnosis of this etiological agent in dogs with gastroenteritis in Ecuador in order to provide prompt and suitable treatment. The assay was specific for amplification of the genome of CaAstV, as no amplification was shown for other canine enteric viruses (CPV-2, CCoV and CDV), sensitive by being able to detect up to one copy of viral genetic material, and repeatable with inter- and intra-assay coefficients of variation of less than 10% between assays. The standard curve showed an efficiency of 103.9%. For the validation of this method, 221 fecal samples from dogs affected with gastroenteritis of various ages from different provinces of Ecuador were used. From the RT-qPCR protocol, 119 samples were found positive for CaAstV, equivalent to 53.8% of the samples processed. CaAstV was detected in dogs where both the highest virus prevalence in the tested strains and the highest viral loads were seen in the younger canine groups up to 48 weeks; in addition, different strains of the virus were identified based on a sequenced fragment of ORF1b, demonstrating the first report of the presence of CaAstV circulating in the domestic canine population affected by gastroenteritis in Ecuador, which could be associated with the etiology and severity of enteric disease.

## 1. Introduction

Dogs are the most common pets that are prone to gastrointestinal conditions that cause vomiting and diarrhea, and in aggravated cases can lead to death; the most susceptible dogs are puppies [1]. Enteric viruses are responsible for more than 60% of the gastrointestinal disorders in dogs worldwide. Among the main enteric viruses in dogs are canine parvovirus (CPV-2), canine distemper virus (CDV), canine adenovirus, canine coronavirus (CCoV), canine rotavirus (CRV) and canine astrovirus (CaAstV). CaAstV stands out for having been repeatedly related in cases of gastrointestinal diseases (GIDs), hepatitis and nephritis [2,3,4].

CaAstV was first described in 1980 in the feces of puppies with diarrhea by electronic microscopy and was fully characterized in 2015 by complete genome sequencing [5]. Belonging to the genus Mamastreovirus and the family Astroviridae, CaAstV is an icosahedral capsid virus, with a positive monocatenary RNA of ~6.6 kb. It has a genome with three open reading frames (ORF1a, ORF1b and ORF2) which partially overlap at their ends. ORF1, located at the 5′ end of the genome, encodes the non-structural proteins, protease and RNA-dependent RNA polymerase (RdRp), and at the 3′ end, ORF2 encodes the capsid protein, considered a hypervariable region that corresponds to the antigenic region of the virus [6,7].

The most severe outbreaks of astrovirus in dogs recorded as the sole causative agent are detailed in young dogs aged 10 days to 3 months with watery diarrhea, vomiting and severe dehydration [5,8]. As these symptoms are multifactorial, the diagnosis of astrovirus is a challenge. Originally, electron microscopy (EM) from feces was used to identify virions; however, certain forms of this virus did not exhibit the distinctive star shape [5]. To facilitate in the diagnosis of this virus, molecular techniques based on endpoint PCR have emerged which are able to detect various types of astroviruses in dogs with gastroenteritis.

CaAstV infections have been GIDs of dogs in Asia, North America, South America, Australia and Europe, mainly in co-infection with CPV, CDV and CCoV [9,10,11,12]. In this research, it has been shown that the genetic structure of CaAstV is prone to recombination. This event plays a crucial role in the development of the virus and its ability to spread. It encompasses the reproduction of the virus in the patient, the heightened pathogenicity and the reduced efficiency of immunity during infection [13,14]. Four phylogenetically differentiable CaAstV lineages have been previously identified and have shown variability in virulence and, therefore, differences in the severity of the disease [15].

CaAstV is an emerging virus, and previous studies have reported that 26% of dogs with gastroenteric signs are positive for this virus. Despite the above, studies and research on CaAstV are limited. Because the distribution of the virus is worldwide, including reports in Latin America, it is necessary to carry out monitoring studies for the presence of this virus in cases of GIDs in canines [16,17,18,19]. The present study conducts a trial for the detection of CaAstV in dogs with gastroenteritis in Ecuador using a RT-qPCR trial based on SYBR Green to detect and quantify viral particles as a first step for the monitoring of this pathogen as a possible etiological agent of gastroenteritis in Ecuador.

## 2. Materials and Methods

### 2.1. Sampling

For this study, 221 samples of feces from dogs of various ages (Supplementary Material File) with gastroenteritis, principally diarrhea, vomiting, fever, loss of appetite and dehydration, were processed. The samples were received at the research laboratories of the Universidad de Las Americas from different provinces of Ecuador [Pichincha (149 samples), Carchi (47 samples) and Imbabura (25 samples)], to determine the causal agent of the digestive symptoms. 

These samples were previously subjected to molecular screening for CPV-2, and were found to be positive for CPV-2. The samples were rescreened for CaAstV by molecular analysis using the RT-qPCR assay presented herein, thus allowing for the RT-qPCR assay to be standardized, validated and able to diagnose the virus for the first time in co-infection in the analyzed samples with CPV-2. 

### 2.2. RNA Extraction

Fecal samples were placed in 2 mL microtubes with 1 mL of Phosphate Saline Buffer (PBS) at 0.1 M, pH 7.4, forming a 1:1 suspension (~0.8 g of feces). The samples were frozen at –80 °C for 10 min, heated to 56 °C for 1 min and homogenized by vortex. This process was repeated three times, then the samples were centrifugated at 12,000× *g* for 30 min at 4 °C. An aliquot of 250 μL of the suspension supernatant was used for the extraction of viral RNA using TRIzol Reagent (Invitrogen by Life Technologies, Carlsbad, CA, USA) according to the manufacturer’s instructions.

### 2.3. Primer Design

**Figure 1:**
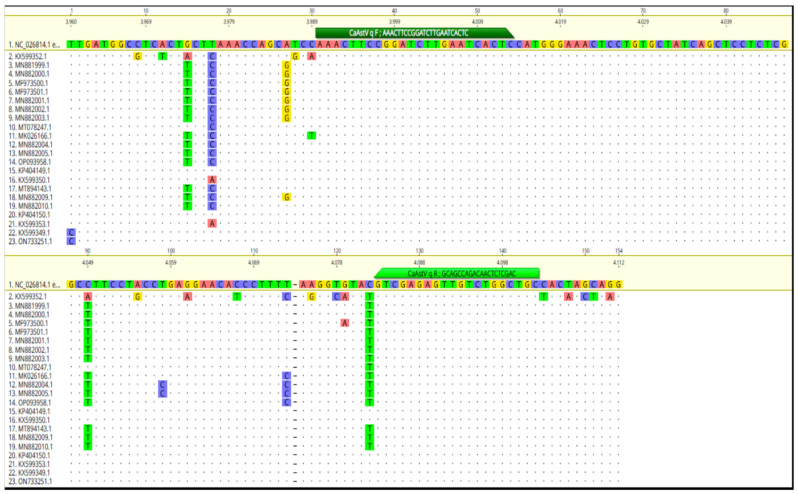
Alignment built with the sequences (NC_026814.1; KP404149.1; KP404150.1; KX599349; KX599350; KX599352.1; KX599353.1; MF973500.1; MF973501.1; MT078247.1; MK026166.1; OP093958.1; MT894143.1; MN881999.1; MN882000.1; MN882001.1; MN882002.1; MN882003.1; MN882004.1; MN882005.1; MN882009.1; MN882010.1 and ON733251.1) used for primer design. The bars in green (the dark green bar in the sequence indicates the forward primer and the aqua green bar indicates the reverse primer) on the reference sequence indicate the location of the primers. The colors of the nucleotides indicate, green = Thymine (T); red = Adenine (A); blue = Cytosine (C); yellow = Guanine (G).

For the present study, the Geneious Prime^®^ 2022.2.2 program [20] was used to design specific primers (Table 1) to amplify a conserved region of the overlapping region of the ORF1b and ORF-2 genes, for which an alignment was made with the complete genome sequences of CaAstV. Sequences for NC_026814.1; KP404149.1; KP404150.1; KX599349; KX599350; KX599352.1; KX599353.1; MF973500.1; MF973501.1; MT078247.1; MK026166.1; OP093958.1; MT894143.1; MN881999.1; MN882000.1; MN882001.1; MN882002.1; MN882003.1; MN882004.1; MN882005.1; MN882009.1; MN882010.1 and ON733251.1 were obtained from GenBank (Figure 1).

### 2.4. Standard Curve Construction

For the construction of the standard curve, a synthetic double-stranded DNA fragment (gBlock Gene Fragments—IDT, Integrated DNA Technologies, Inc., Coralville, IA, USA) was designed that contains the target sequence that the designed primers amplify (nucleotide position 3990–4102 in NC_026814.1). The DNA Copy Number and Dilution Calculator (Thermo Fisher Scientific, Carlsbad, CA, USA) computer tool was used to determine the amount of gBlock needed to produce a dilution with a concentration of 10^8^ copies × µL. From this, a 10-fold serial dilution was carried out until a copy was obtained. These standards were also used for determining the Limit of Detection (LoD) and Limit of Quantification (LoQ).

### 2.5. RT-qPCR for CaAstV Detection

The extracted RNA (8 µL) was subjected to a reverse transcription reaction using SuperScriptTM III Reverse Transcriptase (Invitrogen, Carlsbad, CA, USA) in accordance with the manufacturer’s instructions, using 1 μL of oligo (dT) 20 and 1 μL of a random hexameric primer. The obtained cDNA was subjected to a qPCR reaction using specific primers for CaAstV detection (Table 1). The reaction contained Power Up SYBR Green Master Mix 2X (Applied Biosystem by Thermo Fisher Scientific, Carlsbad, CA, USA), 0.8 μM per primer, 1 μL of sample cDNA and UltraPure DNase/RNase-Free Distilled Water H_2_O (Invitrogen, Carlsbad, CA, USA) needed to bring the reaction contents up to 10 μL. NTCs (No Template Controls) were prepared by substituting the cDNA with an equal volume of dH_2_O. The reaction was carried out in a CFX96 Touch Real-Time PCR Detection System (Bio-Rad Laboratories, Inc., Hercules, CA, USA) in fast mode with the following conditions: a UDG activation step at 50 °C for 2 min, a Dual-Lock DNA polymerase step at 95 °C for 2 min, followed by 40 cycles of 95 °C for 3 s and 60 °C for 30 s. A melting curve was performed in all runs. All samples were tested in duplicate, and absolute quantification was performed relative to the standard curve used in each run. A synthetic double-stranded DNA fragment (gBlock) was used as the positive control and ddH_2_O was used as the negative control (NTC) for each assay to ensure functionality. 

### 2.6. Specificity of RT-qPCR Test

Positive samples for CPV-2, CCoV and CDV were used to determine the specificity of the test. These were subjected to the developed RT-qPCR test in order to not produce amplifications with genetic material different from CaAstV, and thus confirm that the test only amplifies samples that are positive for the studied virus.

### 2.7. Repeatability of Assay

To assess the intra-assay and inter-assay repeatability and stability of the RT-qPCR assay, five serial dilutions with base 10 of the synthetic double-stranded DNA fragment containing the target genomic sequences of CaAstV (gBlock) were prepared for the assay of RT-qPCR; each of these dilutions was aliquoted separately and stored at −20 °C until use. The mean Cq value and the coefficient of variation (CV) were calculated according to the test results, and the stability of the assay was assessed by the CV. 

Inter-assay repeatability: For inter-assay repeatability, an aliquot of each of the five serial dilutions described above was used and amplified by RT-qPCR five times under the same reaction conditions. In each amplification, a different aliquot of the five serial dilutions was thawed and checked for variations in the Cq from 0.5 to 1 at the points of the curve of each thawed aliquot during each amplification. 

Intra-assay repeatability: five serial dilutions with base 10 of the synthetic double-stranded DNA fragment containing the target genomic sequences of CaAstV were taken, and 5 replicates were made for each dilution factor. The RT-qPCR assay was performed at the same time.

### 2.8. PCR End Point and Sanger Sequencing

To confirm the presence of CaAstV in the analyzed samples, 10 samples considered positive by the present assay were randomly selected and subjected to an endpoint heminested PCR for amplification of a fragment of RdRp of ~400 pb in ORF1b using primers previously reported by Chu in 2008 [21]. For this, 3 µL of cDNA from the previously mentioned PCR was placed in a Master Mix containing 2.5 µL of 10X Buffer, 0.5 µL of 10 mM dNTPs, 1 µL of 50 mM MgCl_2_, 1 U of Platinum Taq DNA polymerase (Invitrogen by Thermo Fisher Scientific), 0.5 µM of each primer (Table 1) and UltraPure™ DNase/RNase-Free Distilled Water dH2O (Invitrogen™ Van Allen Way, Carlsbad, CA, USA) to bring the solution up to 25 µL. The PCR protocol included a step of 95 °C for 5 min and 35 cycles of 30 s at 95 °C, 30 s at 50 °C and 45 s at 72 °C, finished by 72 °C for 10 min; the same protocol was followed in both steps of the heminested PCR. The generated amplicons were purified using ExoSAP-IT™ Express PCR Product Cleanup (Applied Biosystems, Santa Clara, CA, USA) following the manufacturer’s instructions. Finally, each purified product was sequenced in the forward and reverse directions using a BigDye^®^ Terminator v3.1 Cycle Sequencing Kit (Thermo Fisher Scientific) and was read on the 3500 Series Genetic Analyzer (Applied Biosystems, Foster City, CA, USA).

### 2.9. Phylogenetic Analysis

The obtained electropherograms were analyzed using Geneious Prime Software version 10.2.4 (Biomatters Ltd., Auckland, New Zealand) and analyzed using BLAST to determine if they were similar to sequences found in GenBank. The obtained sequences were sequenced with other sequences downloaded from GenBank using Clustal Omega V1.2.2 software. A Maximum Likelihood tree was created using RAxML (v8.2.10) based on the GTR GAMMA nucleotide model and with the Bootstrap test algorithm with 1000 repeats, using a previously reported method for the analysis of CaAstV sequences [15].

### 2.10. Statistical Analysis

In the present study, descriptive statistics of samples were carried out. The Shapiro–Wilk test was used to analyze whether samples had a normal distribution, and Fisher X2 tests were used to analyze whether age was a statistically significant factor for the presence of the virus. A Kruskal–Wallis H test was run to determine the variations between the viral copies quantified for the analyzed groups. All these analyses were performed in the Jamovi 2.2.5 statistical program using 95% confidence.

### 2.11. GenBank Accession Numbers

The nucleotide sequences of a portion of the ORF 1b gene identified in the present study were deposited in GenBank under the following accession numbers: PP889830.1 (283D ECU), PP889831.1 (371D ECU), PP889832.1 (370D ECU), PP889833.1 (380D ECU), PP889834.1 (442D ECU), PP889835.1 (286D ECU), PP889836.1 (288D ECU), PP889837.1 (255D ECU), PP889838.1 (366D ECU) and PP889839.1 (393D ECU).

## 3. Results

### 3.1. Standard Curve

**Figure 2:**
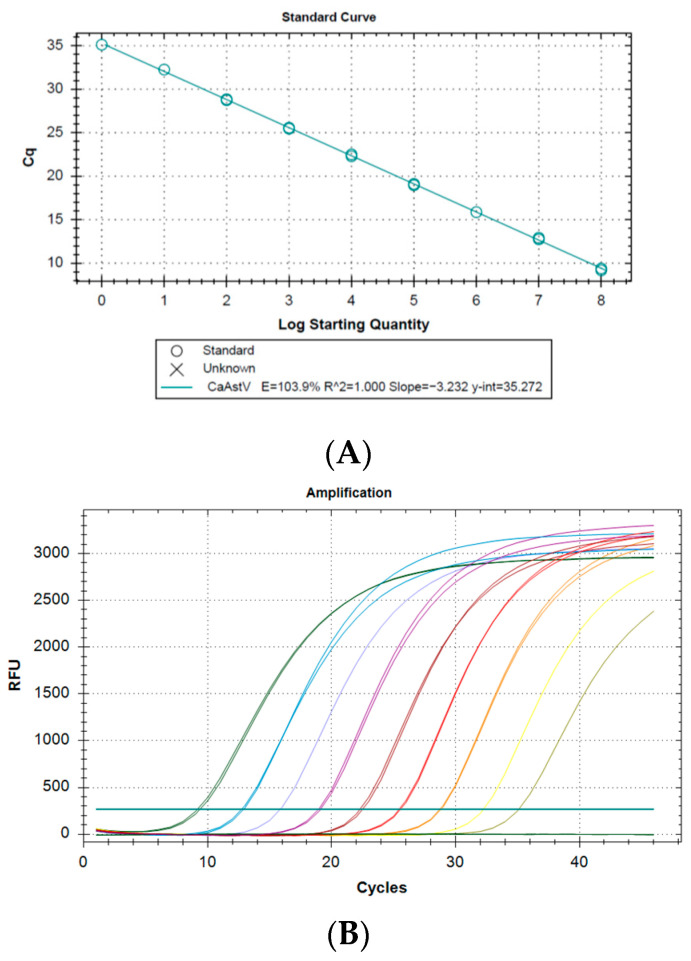

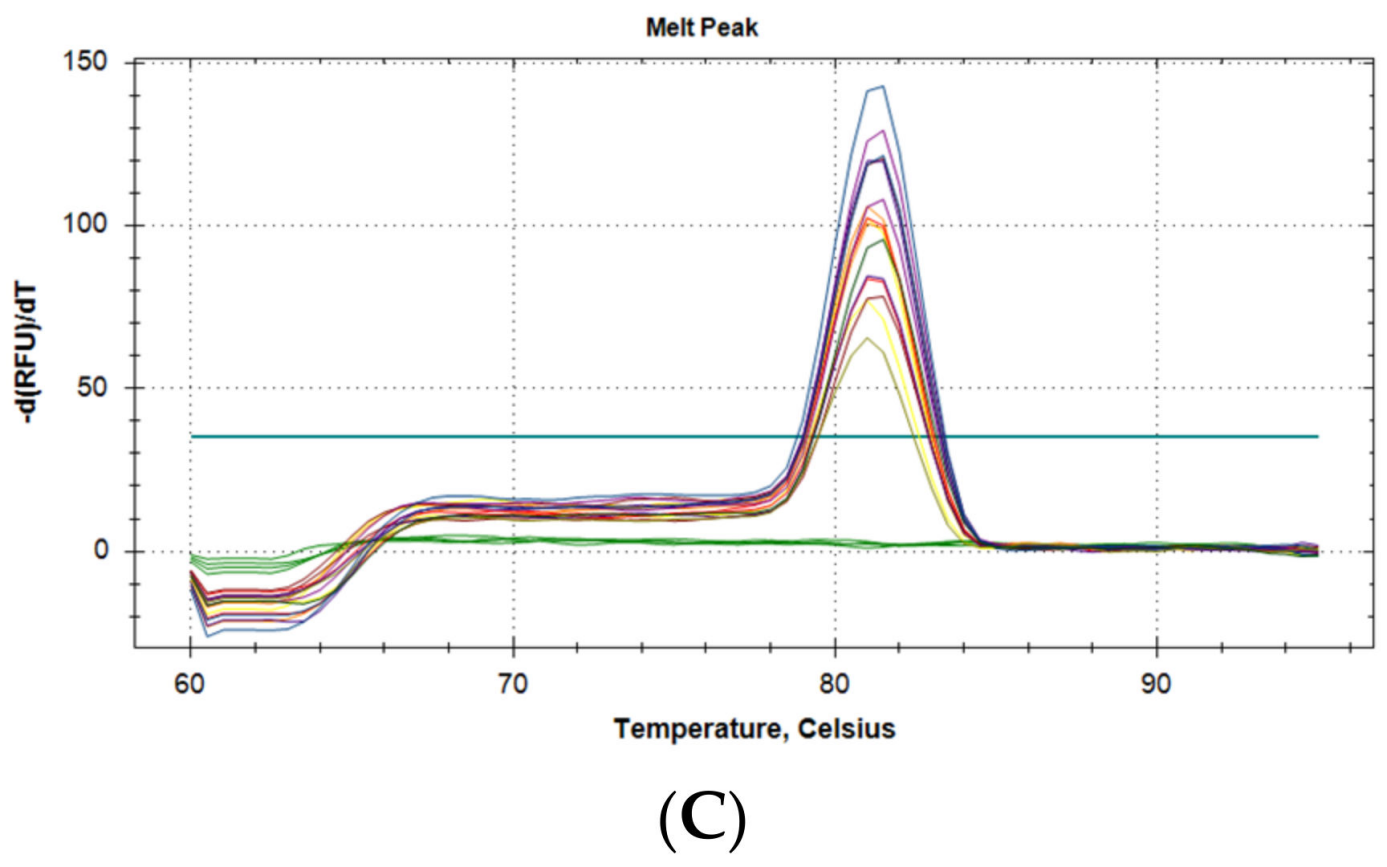
Test for determining the calibration curve for the quantification of CaAstV using SYBR Green-based qPCR: (**A**) standard curve, (**B**) amplification plot and (**C**) melting curve. Each color represents a dilution used in the standard curve.

The 10-fold serial dilution (10^8^ to 10^0^) of the synthetic double-stranded DNA fragment containing the target genomic sequences of CaAstV (gGBlock) generated a standard curve with an efficiency of 103.9%, a slope of −3.232 and a correlation coefficient of 1.000. (Figure 2A). The amplification plot (Figure 2B) for each of the points allowed for the determination of both the LoD and LoQ, showing that these values were up to 10^0^ copies/µL of genetic material. All amplified points showed a melting curve at 81.5 °C, corresponding to the fragment of interest (Figure 2C), and no dimers or non-specific products were present in any run.

### 3.2. Specificity of qPCR Assay

The RT-qPCR specificity trial for CaAstV detection only showed amplification curves for samples positive for CaASTV. No amplification was observed in any of the positive controls of CPV-2, CCoV or CDV.

### 3.3. Repeatability of Assay

The repeatability test carried out using different dilutions of gBlock, with amounts ranging from 10^8^ to 10^4^ copies, showed a fluctuation in the coefficient of variation between assays, ranging from 0.109% to 0.910%. Likewise, variability in the coefficient of variation within assays was observed, with values ranging from 0.346% to 0.983% (Table 2).

### 3.4. RT-qPCR for CaAstV Detection

**Figure 3:**
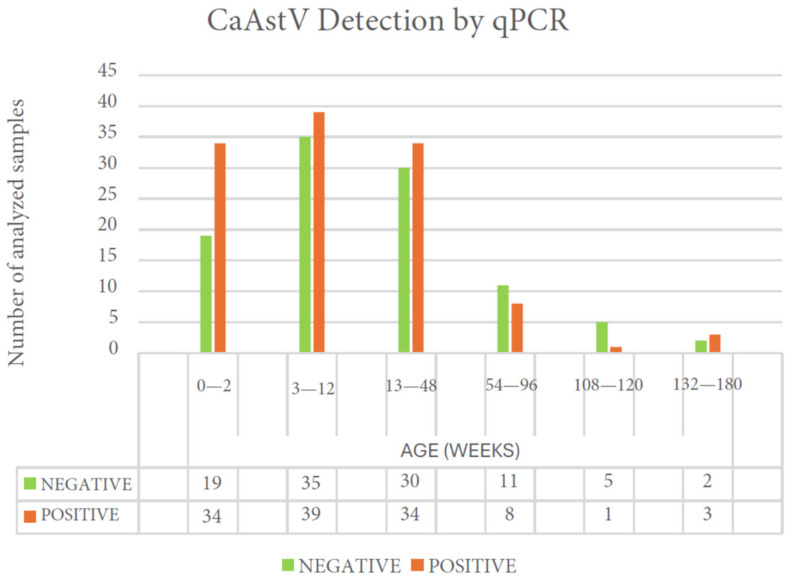
CaAstV detection in dogs with gastroenteritis that were CPV-2-positive according to the age of the animals.

From the total of 221 samples from dogs with gastroenteritis, 119 samples were positive for CaAstV, corresponding to 53.8% of the processed samples, and 102 samples were negative, corresponding to 46.2% of the samples. In Pichincha, 94 samples were positive for CaAstV, in Carchi there were 24 positive samples, and there was only 1 positive sample in Imbabura. The samples were separated into age groups (by week) as shown in Figure 3. The assay showed a higher presence of CaAstV compared to the total number of samples in the groups spanning 54 to 96 weeks and 108 to 120 weeks relative to the corresponding total samples. The groups with the highest number of positive samples were the youngest groups up to 48 weeks; however, the percentage of positivity in these groups was less than 50% for each group. The Shapiro–Wilk test showed that the analyzed samples had a non-normal distribution (*p*-value < 0.001), and the Fisher’s exact test showed that age had no significance on the presence of the disease in the set of samples that was used (*p*-value = 0.237).

The RT-qPCR assay allowed for quantification of the viral load in each of the CaAstV-positive samples used in this study. Samples grouped on the basis of age showed the highest average viral load in the 3–12 weeks of age group, being from the youngest individuals, and the lowest average load was in the individuals at 132–180 weeks of age, corresponding to the longest-lived individuals (Table 3). A single individual at 2–12 weeks of age showed the highest viral load, and two individuals’ samples showed viral loads of 10^0^ GCs/~0.2 mg of extracted feces according to the dilutions. However, none of the tested groups showed significant differences between them (*p*-value > 0.05).

### 3.5. Phylogenetic Analysis

**Figure 4:**
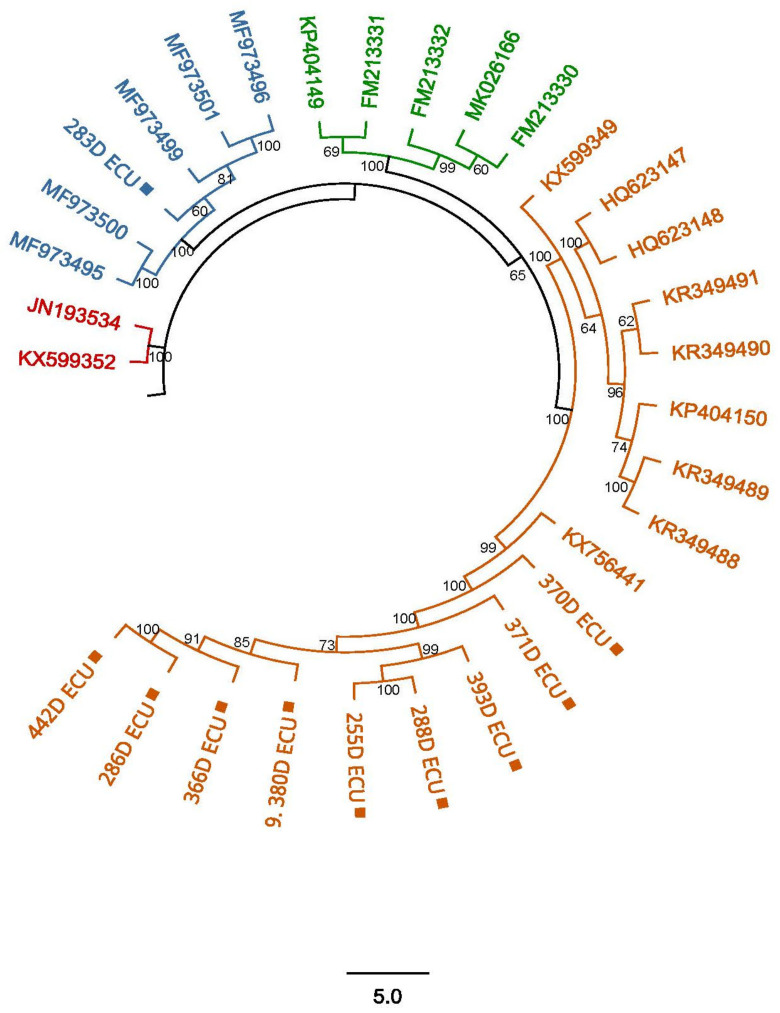
Phylogenetic analysis between CaAstV sequences obtained in this study and other sequences collected from NCBI from Australia, Brazil, the United Kingdom, Italy, Hungary and China, based on NT fragments of ORF1b, created using an ML method using the RAxML with the GTR + GAMMA model, a 1000 bootstrap and a threshold value of 70%. The sequences are grouped and identified by color based on the lineage to which they belong, with red, blue, green and orange for lineages 1, 2, 3 and 4, respectively. Sequences obtained in this study are marked with ■. The bootstrap percentage is marked in all nodes.

The sequences obtained in the present study showed a high similarity of nucleotides (NTs) to other sequences deposited in GenBank corresponding to CaAstV, confirming the presence of this virus in dogs with gastroenteritis in Ecuador. The phylogenetic tree generated clades corresponding to the four lineages previously described by Zhang in 2020 [15]. Samples 380D, 366D, 286D, 393D, 288D, 255D, 371D and 370D were grouped in the clade corresponding to lineage 4, with sequences reported in China, Brazil, the United Kingdom, Hungary and Italy; sample 283D was grouped with samples corresponding to lineage 2, with sequences reported in Australia and China (Figure 4).

When analyzing the similarity of nucleotides between the sequenced samples and the sequences collected from GenBank, it was observed that from one lineage to the other, the sequences have a similarity of only between 40% and 60%, while between sequences of the same lineage, these have a similarity of more than 85%. In some cases, the differences are more drastic, as can be seen for sample 283D, grouped in lineage 2, and sequence JN193534, corresponding to lineage 1, which are only 30.576% similar (Table 4).

The analysis of the percentage of nucleotide similarity between the sequences obtained here and others from other countries showed that in lineage 2, the 283D ECU sample has a 73.7–97.8% nucleotide similarity with sequences from China; on the other hand, when comparing the rest of the sequences generated here and grouped in lineage 4, they showed a nucleotide similarity of 67.4–96% with a sequence from Australia, and a nucleotide similarity of 66.9–96.1% with the sequences from Brazil. This great variability in the percentage of nucleotide similarity could be due to the high recombination rate that the Orf 1b gene presents [22], and denotes that CaAstV genotyping cannot be performed based on nucleotide similarity, and thus a phylogenetic analysis must be performed to observe how the sequences are grouped in each group or lineage [15].

**Table 4:** Comparison between the nucleotides of CaAstV sequences obtained in this study and the sequences collected from NCBI.

Comparation of Sequences by Lineages																					
Lineage	N°	Sequence	1	2	3	4	5	6	7	8	9	10	11	12	13	14	15	16	17	18	19
**1**	**1**	JN193534 ITA	-	99.3%	76.2%	74.5%	75.2%	76.5%	73.9%	75.9%	75.9%	75.0%	62.4%	53.4%	81.4%	75.3%	74.1%	74.9%	75.9%	75.6%	63.6%
	**2**	KX599352 HUN	99.3%	-	74.6%	73.7%	73.9%	75.2%	73.1%	74.0%	74.7%	73.8%	63.7%	56.7%	77.6%	74.5%	73.5%	73.4%	74.5%	74.7%	65.0%
**2**	**3**	283D ECU ■	76.2%	74.6%	-	95.4%	95.3%	93.2%	94.5%	94.5%	94.1%	95.6%	94.1%	80.6%	90.0%	93.8%	95.0%	95.0%	93.8%	93.8%	90.0%
	**4**	MF973500 CH	74.5%	73.7%	95.4%	-	97.8%	95.0%	95.1%	95.0%	95.1%	95.0%	82.1%	68.2%	82.1%	95.2%	95.0%	95.0%	95.1%	94.4%	81.5%
	**5**	MF973501 CH	75.2%	73.9%	95.3%	97.8%	-	94.4%	95.1%	94.2%	95.0%	95.1%	81.8%	67.6%	95.0%	95.0%	95.2%	95.1%	95.0%	94.1%	81.2%
**3**	**6**	KP404149 UK	76.5%	75.2%	93.2%	95.0%	94.4%	-	96.4%	94.4%	95.9%	94.9%	81.5%	67.9%	95.5%	94.4%	94.7%	94.6%	95.9%	98.7%	79.4%
	**7**	MK026166 AUS	73.9%	73.1%	94.5%	95.1%	95.1%	96.4%	-	94.2%	94.1%	95.6%	81.8%	67.6%	95.9%	94.4%	95.5%	95.4%	94.1%	94.6%	81.0%
**4**	**8**	370D ECU ■	75.9%	74.0%	94.5%	95.0%	94.2%	94.4%	94.2%	-	95.5%	95.6%	94.5%	81.3%	95.9%	94.2%	94.6%	94.8%	94.1%	96.1%	94.7%
	**9**	KX756441 AUS	75.9%	74.7%	94.1%	95.1%	95.0%	95.9%	94.1%	95.5%	-	95.3%	81.0%	67.4%	96.0%	95.2%	96.3%	96.2%	97.4%	95.7%	80.2%
	**10**	380D ECU ■	75.0%	73.8%	95.6%	95.0%	95.1%	94.9%	95.6%	95.6%	95.3%	-	98.9%	85.2%	98.9%	94.3%	95.8%	95.6%	95.0%	95.3%	95.6%
	**11**	442D ECU ■	62.4%	63.7%	94.1%	82.1%	81.8%	81.5%	81.8%	94.5%	81.0%	98.9%	-	78.8%	98.4%	80.6%	81.7%	81.5%	80.8%	81.8%	87.2%
	**12**	286D ECU ■	53.4%	56.7%	80.6%	68.2%	67.6%	67.9%	67.6%	81.3%	67.4%	85.2%	78.8%	-	98.2%	67.0%	67.8%	67.6%	66.9%	67.6%	72.9%
	**13**	288D ECU ■	81.4%	77.6%	90.0%	82.1%	95.0%	95.5%	95.9%	95.9%	96.0%	98.9%	98.4%	98.2%	-	94.3%	95.7%	95.5%	94.7%	95.5%	95.1%
	**14**	KR349491 BRA	75.3%	74.5%	93.8%	95.2%	95.0%	94.4%	94.4%	94.2%	95.2%	94.3%	80.6%	67.0%	94.3%	-	95.5%	95.4%	97.2%	93.9%	80.0%
	**15**	KR349488 BRA	74.1%	73.5%	95.0%	95.0%	95.2%	94.7%	95.5%	94.6%	96.3%	95.8%	81.7%	67.8%	95.7%	95.5%	-	99.8%	96.3%	94.5%	81.1%
	**16**	KR349489 BRA	74.9%	73.4%	95.0%	95.0%	95.1%	94.6%	95.4%	94.8%	96.2%	95.6%	81.5%	67.6%	95.5%	95.4%	99.8%	-	96.2%	94.4%	81.0%
	**17**	KR349490 BRA	75.9%	74.5%	93.8%	95.1%	95.0%	95.9%	94.1%	94.1%	97.4%	95.0%	80.8%	66.9%	94.7%	97.2%	96.3%	96.2%	-	95.6%	80.0%
	**18**	KX599349 BRA	75.6%	74.7%	93.8%	94.4%	94.1%	98.7%	94.6%	96.1%	95.7%	95.3%	81.8%	67.6%	95.5%	93.9%	94.5%	94.4%	95.6%	-	79.2%
	**19**	371D ECU ■	63.6%	65.0%	90.0%	81.5%	81.2%	79.4%	81.0%	94.7%	80.2%	95.6%	87.2%	72.9%	95.1%	80.0%	81.1%	81.0%	80.0%	79.2%	-
					**% of similarity by nucleotides**																

Sequences with ~100% nucleotide similarity were removed (393D ECU, 255D ECU and 366D ECU). Sequences obtained in this study are marked with ■. ITA = Italy, HUN = Hungary, UK = United Kingdom, AUS = Australia, BRA = Brazil, ECU = Ecuador.

## 4. Discussion 

Gastrointestinal diseases in dogs are common and of clinical interest, as they have multifactorial origins, including bacterial, parasitic or viral [9,23,24]. However, diarrheas caused by bacteria and parasites have faster and cheaper diagnostic methods, including direct fecal smear with or without gram staining or direct fecal smear (coproparasitoscopic). In the case of viral diseases, the most common method is the detection of viral antigens that, depending on the sensitivity and specificity of the test, can show false positive or false negative results. Although CPV-2 is usually attributed as the most common cause of viral gastroenteritis in dogs, the recent accelerated distribution of CaAstV around the world and the incidence of GIDs have brought it into focus as a pathogen of clinical interest. The present study focused on the diagnosis of CaAstV by standardization and development of a RT-qPCR assay based on SYBR Green for the detection and quantification of CaAstV in dogs with gastroenteritis in Ecuador to identify the presence of this virus as an emerging pathogen that causes GIDs [6,7,25]. 

The study showed that the RT-qPCR assay was sensitive by having the capacity to amplify up to one copy of the viral genetic material (Figure 2), and specific by not showing amplification in the presence of other gastroenteritis-causing viruses. Fluorescence-based real-time qPCRs are currently the main form of diagnostics, compared to LAMP-based methods that require more customized designs and are prone to contamination. Both TaqMan-based qPCR and SYBR Green-based qPCR methods provide the possibility of quantification of genetic material by detection of emitted fluorescence; in this case, a SYBR Green-based method was chosen due to the high sensitivity it provides. In addition, by using a fast protocol, the reaction time during amplification was reduced compared to standard RT-qPCR. Other molecular assays based on hydrolysis probes also showed efficient and fast CaAstV detection, but the amplification capacity was up to 10^1^ copies/uL of viral genetic material [26,27]. This study provides the first evidence of CaAstV circulation in dogs with gastroenteritis in Ecuador. CaAstV is a virus that has been linked to increased canine mortality, particularly in puppies and when co-infected with other enteric viruses such as CPV-2. Previous studies have already identified CPV-2 in canines in Ecuador [28].

CaAstV was detected and quantified in stool samples from dogs of all ages and from different provinces of Ecuador, showing a higher number of positive cases in samples from dogs under 48 weeks of age (Figure 3), which implies a risk for these canines given the high mortality in cases of gastroenteritis [1,29,30]. In the RT-qPCR assay, 58.3% of the 221 processed samples were found to be positive for CaAstV, in contrast to other reports in Latin America where only 25.3% of the samples were positive in dogs with symptoms of gastroenteritis out of a total of 253 samples. However, to ensure the analysis of the incidence rate of this pathogen in dogs with gastroenteritis in Ecuador, it is necessary to carry out a more extensive assessment using a larger number of samples covering a more considerable area of the country. The quantification of CaAstV viral loads in samples from dogs with gastroenteritis showed higher average viral loads in dogs aged 3 to 12 weeks (Table 3), corresponding to periods of loss of innate immunity provided by the mother, which would increase susceptibility to infection and development of aggravated disease and result in an elevated probability of mortality in puppies [29,31].

After analysis of the ORF1b fragment sequences, it was observed that they showed a similarity between 67% and 97% with respect to other CaAstV sequences deposited in GenBank, corresponding to the four lineages of the virus previously reported [13,15,32]. Although ORF1b is considered a conserved gene in each group of mamastroviruses, the sequences reported among CaAstV lineages show a high variability among them, being only 62% similar in some cases (Table 4). Nevertheless, although ORF1b clustered sequences in the previously described lineages, the circulation of these variants (2 and 4 lineages) in the country could not be fully assured by the need to conduct CaAstV genotyping specifically targeting the ORF2 gene, hence more studies must be carried out to confirm the presence of these CaAstV variants in Ec-uador and he phylogenetic relationship that they could have with the sequences from China (lineage 2) and with the sequences from Australia and Brazil (lineage 4)Although vertical transmission of astroviruses in mammals has not been documented [33], astrovirus infections have been shown to contribute to aggravated gastrointestinal disease in young dogs and is compounded by the absence of vaccines, which makes epidemiological control more difficult. Previous studies have detailed that this virus has a tendency to recombine with other astroviruses, as well as a high mutability rate that increases the virulence of this pathogen [34,35]. However, these studies are based on ORF2, and since it encodes the capsid protein, the changes generated in this protein directly influence the infection capacity and susceptibility to previous immunity in canines; therefore, it is necessary to continue with research based on this emerging virus in Ecuador and other countries as a cause of GIDs [36,37]. To our knowledge, the present study is the first to demonstrate the presence of CaAstV in dogs with gastroenteritis in Ecuador. This virus may be the main cause of disease or it may operate in conjunction with other enteric viruses like CPV-2, CDV, CCoV or non-classical enteric viruses such as canine kobuvirus or canine circovirus, perhaps worsening the severity of the illness. The age of canines in which CaAstV or co-infection with classical or non-classical enteric viruses provokes gastrointestinal infection and which viral combination is more prevalent and is associated with the severity of the disease needs to be investigated. For this reason, more studies need to be carried out to determine the involvement of the astrovirus in enteric diseases in dogs in Ecuador, and in this way show veterinary doctors that other viral agents could be affecting the health of pets, and that using a rapid, sensitive and specific diagnostic method, as demonstrated here for the detection of CaAstV, could enable the implementation of suitable measures and therapies to reduce the severity of gastrointestinal disorders and, most importantly, prevent the mortality of pets.

## 5. Conclusions

This investigation reveals the presence of CaAstV in dogs with gastrointestinal disease in Ecuador for the first time. The study shows that the virus is circulating in dogs of various ages, from young to old animals. Additionally, the dogs in this study were found to be co-infected with CPV-2. These findings suggest that CaAstV may play a role in the development of gastroenteric diseases or may contribute to the severity of the disease. Thus, further research is needed to determine the role of CaAstV as a causative agent of enteric disease in dogs. Additionally, the RT-qPCR assay showed greater sensitivity and specificity for CaAstV detection and quantification.

## Figures and Tables

**Table 1:** Primers used in this study.

Primers	Target	Sequence	Assay	Length
**CaAstV qLN F**	**ORF1b + ORF2** **(Overlapping region)**	AAACTTCCGGATCTTGAATCACTC	RT-qPCR	113 pb
**CaAstV qLN R**		GCAGCCAGACAACTCTCGAC		
				
**PAN ASTRO F1**	ORF1b	GARTTYGATTGG RCKCGKTAYGA	HN-PCR	~400 pb
**PAN ASTRO F2**		GARTTYGATTGGRCKAGGTAYGA		
**PAN ASTRO R**		GGYTTKACCCACATNCCRAA		
**PAN ASTRO HN F1**		CGKTAYGATGGKACKATHCC		
**PAN ASTRO HNF2**		AGGTAYGATGGKACKATHCC		

**Table 2:** Repeatability assays using gBlock dilutions from 10^8^ to 10^4^ copies of genetic material.

Copy Number	Inter-Assay		Intra-Assay	
	Cq Mean	Cq St Dev	Cq Mean	Cq St Dev
10^8^	14.179	0.293	14.248	0.983
10^7^	17.864	0.203	17.329	0.346
10^6^	21.312	0.394	21.634	0.235
10^5^	25.973	0.910	24.932	0.487
10^4^	29.109	0.109	28.752	0.518

Cq = Cycle quantification.

**Table 3:** Summary of quantifications of gene copies (GCs) of CaAstV ordered by ages.

Quantification of CaAstV by qPCR			
Age Groups (Weeks)	Average GCs	Maximum GCs	Minimum GCs
0–2	2.97 × 10^4^	4.46 × 10^5^	5
3–12	1.17 × 10^6^	2.27 × 10^7^	1
13–48	5.83 × 10^4^	3.16 × 10^5^	6
54–96	4.00 × 10^5^	1.26 × 10^7^	1
108–120	2.66 × 10^4^	4.60 × 10^5^	4
132–180	7.73 × 10^3^	1.23 × 10^5^	6

## Data Availability

This article contains all of the data that were created or analyzed throughout the investigation.

## Supplementary Materials

The following supporting information can be downloaded at: https://www.mdpi.com/article/10.3390/vetsci11070303/s1, Table S1: Hoja1.

## References

[1] Unterer S., Busch K. (2021). Acute Hemorrhagic Diarrhea Syndrome in Dogs. Vet. Clin. N. Am. Small Anim. Pract..

[2] Van Nguyen T., Piewbang C., Techangamsuwan S. (2023). Genetic Characterization of Canine Astrovirus in Non-Diarrhea Dogs and Diarrhea Dogs in Vietnam and Thailand Reveals the Presence of a Unique Lineage. Front. Vet. Sci..

[3] Zhu A.L., Zhao W., Yin H., Shan T.L., Zhu C.X., Yang X., Hua X.G., Cui L. (2011). Isolation and Characterization of Canine Astrovirus in China. Arch. Virol..

[4] Dandrieux J.R.S. (2016). Inflammatory Bowel Disease versus Chronic Enteropathy in Dogs: Are They One and the Same?. J. Small Anim. Pract..

[5] Williams F.P. (1980). Astrovirus-like, Coronavirus-like, and Parvovirus-like Particles Detected in the Diarrheal Stools of Beagle Pups. Arch. Virol..

[6] Martella V., Moschidou P., Lorusso E., Mari V., Camero M., Bellacicco A., Losurdo M., Pinto P., Desario C., Bányai K. (2011). Detection and Characterization of Canine Astroviruses. J. Gen. Virol..

[7] Toffan A., Jonassen C.M., De Battisti C., Schiavon E., Kofstad T., Capua I., Cattoli G. (2009). Genetic Characterization of a New Astrovirus Detected in Dogs Suffering from Diarrhoea. Vet. Microbiol..

[8] Kurtz J.B., Lee T.W. (1987). Astroviruses: Human and Animal. Ciba Found. Symp..

[9] Dema A., Tallapally M.R., Ganji V.K., Buddala B., Kodi H., Ramidi A., Yella N.R., Putty K. (2023). A Comprehensive Molecular Survey of Viral Pathogens Associated with Canine Gastroenteritis. Arch. Virol..

[10] Saltık H.S. (2023). Concomitant Virus-Induced Gastrointestinal Infection in Dogs. Pol. J. Vet. Sci..

[11] Wang Y., Li Y., Cui Y., Jiang S., Liu H., Wang J., Li Y. (2021). Duplex SYBR Green I-Based Real-Time PCR Assay for the Rapid Detection of Canine Kobuvirus and Canine Astrovirus. J. Virol. Methods.

[12] Wang Y., Li Y., Cui Y., Jiang S., Liu G., Wang J., Li Y. (2020). Establishment of a Duplex SYBR Green I-Based Real-Time Polymerase Chain Reaction Assay for the Rapid Detection of Canine Circovirus and Canine Astrovirus. Mol. Cell Probes.

[13] Li M., Yan N., Ji C., Wang M., Zhang B., Yue H., Tang C. (2018). Prevalence and Genome Characteristics of Canine Astrovirus in Southwest China. J. Gen. Virol..

[14] Mihalov-Kovács E., Martella V., Lanave G., Bodnar L., Fehér E., Marton S., Kemenesi G., Jakab F., Bányai K. (2017). Genome Analysis of Canine Astroviruses Reveals Genetic Heterogeneity and Suggests Possible Inter-Species Transmission. Virus Res..

[15] Zhang W., Wang R., Liang J., Zhao N., Li G., Gao Q., Su S. (2020). Epidemiology, Genetic Diversity and Evolution of Canine Astrovirus. Transbound. Emerg. Dis..

[16] Caddy S.L., Goodfellow I. (2015). Complete Genome Sequence of Canine Astrovirus with Molecular and Epidemiological Characterisation of UK Strains. Vet. Microbiol..

[17] de Deus D.R., Siqueira J.A.M., Teixeira D.M., Maués M.A.C., de Figueiredo M.J.d.F.M., Sousa E.C., Portal T.M., Soares L.D.S., Resque H.R., da Silva L.D. (2023). Nearly Complete Genome Sequences of Two Canine Mamastrovirus 5 Strains from Latin America. Microbiol. Resour. Announc..

[18] He H.-J., Zhang W., Liang J., Lu M., Wang R., Li G., He J.-W., Chen J., Chen J., Xing G. (2020). Etiology and Genetic Evolution of Canine Coronavirus Circulating in Five Provinces of China, during 2018–2019. Microb. Pathog..

[19] Lizasoain A., Tort L.F.L., García M., Gómez M.M., Leite J.P.G., Miagostovich M.P., Cristina J., Berois M., Colina R., Victoria M. (2015). Sewage Surveillance Reveals the Presence of Canine GVII Norovirus and Canine Astrovirus in Uruguay. Arch. Virol..

[20] Dotmatics Geneious. https://www.geneious.com.

[21] Chu D.K.W., Poon L.L.M., Guan Y., Peiris J.S.M. (2008). Novel Astroviruses in Insectivorous Bats. J. Virol..

[22] Roach S.N., Langlois R.A. (2021). Intra-and Cross-Species Transmission of Astroviruses. Viruses.

[23] Cevidanes A., Di Cataldo S., Muñoz-San Martín C., Latrofa M.S., Hernández C., Cattan P.E., Otranto D., Millán J. (2023). Co-Infection Patterns of Vector-Borne Zoonotic Pathogens in Owned Free-Ranging Dogs in Central Chile. Vet. Res. Commun..

[24] Ojeda-Chi M.M., Rodriguez-Vivas R.I., Esteve-Gasent M.D., Pérez de León A.A., Modarelli J.J., Villegas-Perez S.L. (2019). Ehrlichia Canis in Dogs of Mexico: Prevalence, Incidence, Co–Infection and Factors Associated. Comp. Immunol. Microbiol. Infect. Dis..

[25] Zhou H., Liu L., Li R., Qin Y., Fang Q., Balasubramaniam V.R., Wang G., Wei Z., Ouyang K., Huang W. (2017). Detection and Genetic Characterization of Canine Astroviruses in Pet Dogs in Guangxi, China. Virol. J..

[26] Wang R., Zhang W., Ye R., Pan Z., Li G., Su S. (2020). One-Step Multiplex TaqMan Probe-Based Method for Real-Time PCR Detection of Four Canine Diarrhea Viruses. Mol. Cell Probes.

[27] Wang Y., Xu L., Noll L., Stoy C., Porter E., Fu J., Feng Y., Peddireddi L., Liu X., Dodd K.A. (2020). Development of a Real-Time PCR Assay for Detection of African Swine Fever Virus with an Endogenous Internal Control. Transbound. Emerg. Dis..

[28] DiGangi B.A., Dingman P.A., Grijalva C.J., Belyeu M., Tucker S., Isaza R. (2019). Prevalence and Risk Factors for the Presence of Serum Antibodies against Canine Distemper, Canine Parvovirus, and Canine Adenovirus in Communities in Mainland Ecuador. Vet. Immunol. Immunopathol..

[29] Bhatta T.R., Chamings A., Vibin J., Alexandersen S. (2019). Detection and Characterisation of Canine Astrovirus, Canine Parvovirus and Canine Papillomavirus in Puppies Using next Generation Sequencing. Sci. Rep..

[30] Turan T., Işıdan H. (2020). Molecular Characterization of Canine Astrovirus, Vesivirus and Circovirus, Isolated from Diarrheic Dogs in Turkey. Iran. J. Vet. Res..

[31] Chastant S., Mila H. (2019). Passive Immune Transfer in Puppies. Anim. Reprod. Sci..

[32] Alves C.D.B.T., Budaszewski R.F., Torikachvili M., Streck A.F., Weber M.N., Cibulski S.P., Ravazzolo A.P., Lunge V.R., Canal C.W. (2018). Detection and Genetic Characterization of Mamastrovirus 5 from Brazilian Dogs. Braz. J. Microbiol..

[33] Martella V., Moschidou P., Buonavoglia C. (2011). Astroviruses in Dogs. Vet. Clin. N. Am. Small Anim. Pr..

[34] Sawant P.M., Waghchaure R.B., Shinde P.A., Palikondawar A.P., Lavania M. (2023). Detection and Molecular Characterization of Animal Adenovirus and Astrovirus from Western Maharashtra, India. Viruses.

[35] Zobba R., Visco S., Sotgiu F., Pinna Parpaglia M.L., Pittau M., Alberti A. (2021). Molecular Survey of Parvovirus, Astrovirus, Coronavirus, and Calicivirus in Symptomatic Dogs. Vet. Res. Commun..

[36] Boros Á., Albert M., Urbán P., Herczeg R., Gáspár G., Balázs B., Cságola A., Pankovics P., Gyenesei A., Reuter G. (2022). Unusual “Asian-Origin” 2c to 2b Point Mutant Canine Parvovirus (Parvoviridae) and Canine Astrovirus (Astroviridae) Co-Infection Detected in Vaccinated Dogs with an Outbreak of Severe Haemorrhagic Gastroenteritis with High Mortality Rate in Hungary. Vet. Res. Commun..

[37] Stamelou E., Giantsis I.A., Papageorgiou K.V., Petridou E., Davidson I., Polizopοulou Z.S., Papa A., Kritas S.K. (2022). First Report of Canine Astrovirus and Sapovirus in Greece, Hosting Both Asymptomatic and Gastroenteritis Symptomatic Dogs. Virol. J..

